# A Review of the Biotechnological Production of Methacrylic Acid

**DOI:** 10.3389/fbioe.2020.00207

**Published:** 2020-03-20

**Authors:** Juliana Lebeau, John P. Efromson, Michael D. Lynch

**Affiliations:** Department of Biomedical Engineering, Duke University, Durham, NC, United States

**Keywords:** methacrylic acid, methyl-methacrylate, fermentation, sustainability, bioprocessing

## Abstract

Industrial biotechnology can lead to new routes and potentially to more sustainable production of numerous chemicals. We review the potential of biobased routes from sugars to the large volume commodity, methacrylic acid, involving fermentation based bioprocesses. We cover the key progress over the past decade on direct and indirect fermentation based routes to methacrylic acid including both academic as well as patent literature. Finally, we take a critical look at the potential of biobased routes to methacrylic acid in comparison with both incumbent as well as newer greener petrochemical based processes.

## Introduction

Methacrylic acid (MA) and its ester (methyl methacrylate, MMA) are primarily polymerized into polymethylmethacrylate (PMMA) which is used in the production of acrylic glass (Dormer et al., 1998). Acrylic glass is used in components of electronics, automobile parts, lights (LEDs), signs, and displays (Brydson, 1999; Nagai and Ui, 2004; Ali et al., 2015)^1^. Notably, PMMA has a high biocompatibility and low acute toxicity enabling use in medical applications (Frazer et al., 2005). MMA pricing ranges from $1.75 to $2.25 /kg^2^^,^^3^^,^^4^ with an annual market that will exceed $8 billion USD by 2025, growing at a rate of 8–9% per year.

This increasing demand for MA is not only due to increased demand for acrylic glass, but also the increasing number of new applications for MMA (Brydson, 1999). MMA will continue to be a critical monomer in the future with currently no equivalent replacement (Ali et al., 2015). As demand will continue to grow, more sustainable methods of production need to be considered. Numerous efforts have been made to increase sustainability and reduce waste in petrochemical processes. Recent advances in chemical processes have enabled alternative petrochemical feedstocks and reduced waste (Johnson et al., 2009; Witczak et al., 2010). Additionally, the International Energy Agency in 2012 designated MA as a suitable target for the design of a bio-based process (Burk and Van Dien, 2016)^1^.

Both petrochemical processes and biobased routes have their own strengths and weaknesses. In this review, we discuss the current states of and recent advances in both petrochemical and biobased routes to MMA. We review different bio-based routes as well as the performance requirements of any biobased process to compete with advanced petrochemical technologies. Lastly, we discuss the potential of future bio-based routes to MMA as well as the key barriers for a bioprocess to compete with petrochemistry including conversion yields and feedstock costs.

## Petrochemical Routes

**Figure 1:**
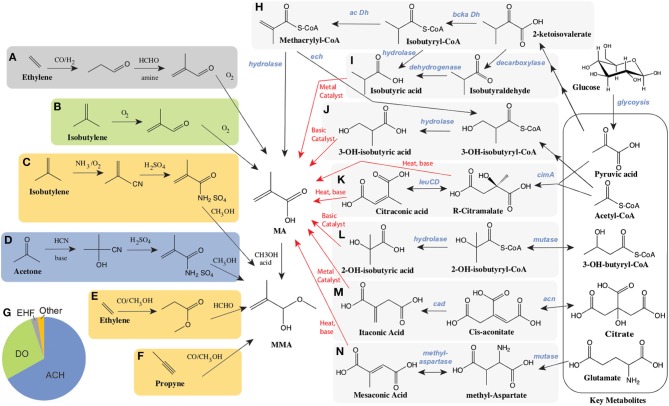
Current petrochemical routes and proposed bioprocessing alternatives for the manufacture of MA/MMA. Petrochemical routes **(A–G)**. The C2 manufacturing routes of MA using ethylene as starting material include **(A)** BASF's ethylene hydroformylation (EHF) to propionaldehyde followed by condensation to methacrolein before oxidation and esterification to MMA and **(E)** the Alpha process where ethylene is converted to MMA *via* methyl propionate. The C3 routes include: **(D)** the Acetone Cyanohydrin (ACH) process starting from acetone and hydrogen cyanohydrin, and **(F)** the propyne route with the carbonylation and esterification of methyl acetylene. The C4 routes, starting from isobutylene include **(B)**, Direct Oxidation (DO) process and **(C)** Asahi's process reliant on ammonia. **(G)** The global breakdown of MMA supply by process technology. Alternative biological routes to produce MA from sugars such as glucose **(H–N)**. Bioconversion steps and chemical conversions are shown as black and red arrows respectively. **(H)** Direct biosynthesis of MA through the intermediates keto-isovalerate, isobutyryl-CoA and methacrylyl-CoA reliant on a branched chain keto-acid dehydrogenase (bcka Dh) and an acyl-CoA dehydrogenase (ac Dh). **(I)** Production of isobutyrate either from keto-isovalerate through isobutyraldehyde, or alternatively through isobutyryl-CoA. **(J)** The conversion of methacrylyl-CoA to 3-hydroxy-isobutyrate (3HIBA) is reliant on an enoyl-CoA hydratase (ech). Citramalate is produced from acetyl-CoA and pyruvate *via* a citramalate synthase (*cimA*) and can then be converted to citraconate *via* isopropylmalate isomerase such as encoded by the *leuCD* genes. Both citramalate and citraconate can be converted chemically to MA. **(L)** 2-hydroxy-isobutyrate (2HIBA) can be produced from 3-hydroxybutyryl-CoA through a mutase and hydrolase. **(M)** Itaconic acid is derived from citric acid through the intermediate cis-aconitate *via* two enzymes: aconitase (acn) and cis-aconitate decarboxylase (cad). **(N)** Mesaconic acid is synthesized from glutamate through methyl-aspartate using a mutase and methylaspartase.

Currently, MMA is produced *via* one of several processes from a few key petrochemical feedstocks as illustrated in Figures 1A–G. Over 65% of MMA is produced *via* the Acetone CyanoHydrin (ACH) route, developed in the 1930's (Figure 1D) (Nagai and Ui, 2004). The use of toxic hydrogen cyanide, as well as concentrated acids are a primary concern with the ACH route, as is the negative impact of significant waste (ammonium bisulfate) generation and treatment (Nagai and Ui, 2004; Mahboub et al., 2018). A competitive route, Direct Oxidation (and similar processes), relies on isobutylene as a feedstock (Figure 1B) and is primarily commercial in Asia (Nagai and Ui, 2004; The Chemical Engineer, 2008; Mahboub et al., 2018). Concerns over safety, costs, and the environmental impact of the ACH route have driven efforts to find alternative routes to produce MMA (Adom et al., 2014). Important among these is the hydroformylation of ethylene and related chemistry (Figures 1A,E). The low costs of ethylene compared to acetone and isobutylene, as well as decreased waste and lower investment costs have made these processes attractive. In 2019, there were announcements related to the potential construction of new MMA plants using ethylene as a feedstock. If constructed these plants would be operational between 2024 and 2026 (Sale, 2019).

## Bioprocessing Alternatives

The use of biotechnology to provide alternative routes for the production of chemicals has had several successful outcomes (Chen et al., 2013; Valdehuesa et al., 2013; Van Dien, 2013; Barton et al., 2015; De Carvalho et al., 2018). Fermentation based routes to MMA utilizing more sustainable feedstocks represent one potential alternative to petrochemistry. Biobased routes may provide both long term environmental and economic sustainability. Given the competitiveness of the market, the development of biobased PMMA has become a priority of many of the current producers (ChiMei Corp., Mitsubishi Corp., Evonik Ind., Sumitomo Chemicals and Arkema) (Bio PMMA Market Trends, 2017)^1^^,^^5^. Previous work on the conversion of sugar to MA or MMA using engineered biocatalysts has focused on the evaluation or development of one of several pathways, as illustrated in Figures 1H–N, ranging from the direct production of MA to the combined use of biochemical and traditional chemistry to produce MA from glucose. In these bio-chemocatalytic routes, key intermediates are produced biologically and subsequently converted to MA. Intermediates evaluated to date include both 2- and 3- hydroxyisobutyric acids, which are converted to MA *via* dehydration, as well as several 5 carbon organic acids (citraconic, citramalic, itaconic, and mesaconic acids), which have the potential to be converted to MA either by decarboxylation or decarboxylation along with dehydration using an inexpensive hot pressurized water process. (Figures 1K,M,N) Significant work over the past decade on these routes has been made in the development of a variety of microorganisms for the biological production of target intermediates, as well as in their subsequent chemical conversions to MA. We will discuss each route in turn.

### Direct Production of Methacrylic Acid

As illustrated in Figure 1H, there is a direct route from glucose to MA. The biochemical steps involved are primarily derived from valine catabolism, *via* the natural intermediate methacrylyl-CoA (Bachhawat et al., 1957; Rendina and Coon, 1957; Massey et al., 1976). In valine catabolism, methacrylyl-CoA is hydrated to 3-hydroxyisobutyryl-CoA, hydrolysed to 3-hydroxyisobutyrate (Figure 1J) and subsequently oxidized to methylmalonate semialdehyde, which enters central metabolism through another oxidation to propionyl-CoA (Bachhawat et al., 1957). Bypassing the later steps of valine degradation by expression of a CoA hydrolase/thioesterase with engineered activity on methacrylyl-CoA, can lead to the direct production of MA. Numerous enzymes have been proposed to perform the conversion of methacrylyl-CoA to MA, which may all need to be engineered for this activity (Burgard et al., 2009). Despite these theoretical descriptions no production has been demonstrated (Burgard et al., 2009). To our knowledge, only one study of microbial bioproduction of MA from glucose was reported reaching a titer of 170uM (14.6mg/L) in shake flasks (Eastham et al., 2018). In this work expression of an acyl-CoA oxidase (ACX4) converts isobutyryl-CoA to methacrylyl-CoA, which is transformed to MA *via* a promiscuous thioesterase (Hayashi et al., 1999; Eastham et al., 2018).

One potential reason for so little success in the biosynthesis of MA is likely due to its acute toxicity. In *E. coli* the concentration of MA reducing growth rate by 50% is only 13.2mM (1.1g/L) (Webb et al., 2018). The toxicity of MA, its esters, and methacryly-CoA has been investigated in numerous *in vivo* studies in both eukaryotes and prokaryotes. The main mechanisms identified were the radical reactivity of MA and derivatives with cellular nucleophiles such as glutathione. MA has also been shown to directly induce DNA damage and inhibit key metabolic enzymes (Plaga et al., 2000; Ansteinsson et al., 2013; Arya et al., 2013; Curson et al., 2014; Murakami et al., 2019). This toxicity makes its direct biological production highly limited in any of the potential microbial hosts considered. (Lipscomb et al., 2011, 2012; Jarboe et al., 2013; Lam et al., 2014; Mukhopadhyay, 2015).

### Isobutyric Acid

As depicted in Figures 1H,I, another route to MA leveraging valine catabolism relies on the biological production of isobutyric acid (IBA), with subsequent oxidation to MA. This route is attractive as significant progress has been made to date on IBA production in engineered hosts. A recent study highlights that *S. cerevisiae* naturally possesses an Ehrlich pathway (Yu et al., 2016), which enables it to produce isobutyrate. However, reported titers of IBA (387.4 mg/L) (Yu et al., 2016) remain relatively low. In contrast, IBA synthesis from glucose in engineered *E. coli* has been reported at titers of 90 g/L, volumetric productivities of 1g/L-h, and yields of 0.39g IBA/g glucose (80% of the theoretical maximum) (Zhang et al., 2011; Xiong et al., 2015). In this work, isobutyraldehyde was first produced from 2-keto-isovalerate using an α-ketoisovalerate decarboxylase (*kivd*) from *Lactococcus lacti*, followed by oxidation to IBA utilizing a phenylacetaldehyde dehydrogenase (*padA*) from *E. coli* (Zhang et al., 2011; Xiong et al., 2015). While these bioprocesses are promising, achieving high chemical conversion yields have proven more challenging. Dehydrogenation has been performed on both IBA to produce MA as well as on methyl-IBA to produce MMA. To date yields of only 40 and 60%, respectively, have been demonstrated. Yields are limited by significant byproduct (carbon dioxide and diisopropyl ketone) formation (McDaniel and Young, 1963; Wilhelm Gruber and Ginter Schröder, 1983; Macho et al., 2004).

### Hydroxy Isobutyric Acids

Routes through biosynthesized hydroxy-isobutyrates (HIBAs) can be coupled with dehydration reactions to produce MA and are considered as alternative chemical conversions to the dehydrogenation of IBA (Figures 1J,L). Both 3-hydroxy-isobutyrate (3-HIBA), derived from valine catabolism as discussed above, as well as its isomer 2-hydroxy-isobutyrate (2-HIBA), have been considered as biological end products (Volker and Schindelmann, 1969; Burgard et al., 2009; Rohwerder and Müller, 2010; Dubois et al., 2011; Burk et al., 2012; Marx et al., 2016). The conversion of 3-HIBA to MA has been reported with conversions from 20 to > 90% (Volker and Schindelmann, 1969; Marx et al., 2016) while the dehydration of 2-HIBA has been accomplished at conversion yields of 71.5% (Pirmoradi and Kastner, 2017). As mentioned above, 3-HIBA is a natural intermediate in valine catabolism. Engineering efforts have resulted in 3-HIBA titers ranging from 150 mg/L (Dellomonaco et al., 2011) to 2.3 g/L (Lang et al., 2015), often produced along with significant amounts of IBA (Lang et al., 2014; Xiong et al., 2015; Jawed et al., 2016; Marx et al., 2016).

2-HIBA-CoA (Figure 1l) was originally found to be a natural metabolite in a pathway that has evolved in the biodegradation of *tert*-butyl ether (Rohwerder et al., 2006). The production of 2-HIBA-CoA *via* a mutase from *A. tertiaricarbonis* is dependent on a B12-dependent mutase involving free radical isomerization (Yaneva et al., 2012; Kurteva-Yaneva et al., 2015). In addition, this enzyme has stereospecificity for (S)-3-hydroxybutyryl-CoA as a substrate (Kurteva-Yaneva et al., 2015). While (R)-3-hydroxybutyryl-CoA, a precursor to polyhydroxyalkanoates, is readily produced from acetoacetyl-CoA in numerous organisms (Madison and Huisman, 1999; Chen and Jiang, 2017), (S)-3-hydroxybutyryl-CoA and (S)-3-hydroxyacyl-CoAs more generally are intermediates in fatty acid biosynthesis. Engineering of pathways with (S)-3-hydroxyacyl-CoA intermediates have been a focus in the production of alcohols as well as fatty acids (Dellomonaco et al., 2011; Lynch et al., 2014; Kim et al., 2015; Wang et al., 2019). 2-HIBA has been produced from both (S)-3-hydroxybutyryl-CoA, *via* mutases similar to that originally characterized, as well as (R)-3-hydroxybutyryl-CoA through the discovery of (R) specific mutases. (R) specific mutases are also vitamin B12 dependent. Biocatalysis reliant on B12 dependent enzymes often require vitamin supplementation, and can suffer from enzyme inactivation, requiring reactivation (Daniel et al., 1998; Mori and Toraya, 1999). To date 2-HIBA biosynthesis has been reported in engineered microbes at titers as high as 6.4g/L (Burgard et al., 2009; Hoefel et al., 2010; Reinecke et al., 2011; Soucaille and Boisart, 2011; Rohde et al., 2017).

### Citramalic/Citraconic Acids

Citramalate is a naturally occurring diacid and an intermediate in the isoleucine biosynthesis pathway of some anaerobic bacteria (Buckel and Barker, 1974; Howell et al., 1999; Risso et al., 2008). Citramalate is synthesized from the central metabolites pyruvic acid and acetyl-CoA using a single enzyme, citramalate synthase (*cimA*), as depicted in Figure 1K. This enzyme has been successfully utilized in pathways enabling the biosynthesis of 1-propanol and 1-butanol through the intermediate citramalate. In these studies directed evolution of citramalate synthase resulted in feedback resistant mutants with improved activity (Atsumi and Liao, 2008). Citramalate has limited toxicity to microbes when compared to MA where concentrations of ~25g/L are required to inhibit growth by 50% (Webb et al., 2018). Citramalate can be converted to MA *via* a relatively simple process involving simultaneous decarboxylation and dehydration with citraconate as an intermediate. The simplest conversion uses only hot pressurized water and has achieved conversion yields as high as 81% (de Jong et al., 2012). Although this chemistry is inexpensive, catalyst development may be needed to improve yields and selectivity. Recent successes in bioengineering highlight the potential of citramalate as an intermediate to MA. Using engineered *E. coli* expressing a mutant citramalate synthase, titers ranging from 46.5 g/L to as high as 80 g/L have been reported with yields of 58% of theoretical (Johnson et al., 2009; Wu and Eiteman, 2016; Parimi et al., 2017; Webb et al., 2018). Additionally, significant systems characterization of these engineered strains has been reported (Webb et al., 2019).

### Itaconic Acid

Itaconic acid has long been produced *via* biotechnology primarily utilizing wild type or engineered *Aspergillus terreus* strains (Steiger et al., 2013; Hevekerl et al., 2014; Bafana and Pandey, 2018; Kuenz and Krull, 2018). Itaconic acid is also produced from citrate through the intermediate cis-aconitate which is decarboxylated to itaconate as illustrated in Figure 1M. Titers in the range of 120–220 g/L have been reported with itaconate yields ranging from 0.45 to as high as 0.58 g itaconic acid/ g glucose and maximal production rates from 0.45 to 1g/L-h (Hevekerl et al., 2014; Huang et al., 2014; Krull et al., 2017a; Tehrani et al., 2019). In addition, the chemical decarboxylation of itaconic acid to MA has been demonstrated *via* several different catalytic routes, mostly reliant on metal catalysts. Some of these processes have demonstrated conversion yields as high as 40% at over 90% selectivity (Le Nôtre et al., 2014; Lansing et al., 2017; Bohre et al., 2019). However, the current cost of itaconic acid ranges from $1.80 to $2.00/kg (Kuenz and Krull, 2018), which is similar to the price of MMA. At this pricing and 100% conversion of itaconic acid to MA (with loss of carbon dioxide), a price of $2.70–$3.00/kg could be expected for MA alone (not including the cost of esterification). This is 20–70% higher than estimated petrochemical based pricing for MA. The route to MA through itaconic acid may well be the most mature, with previous scale up and commercial production but key improvements to reduce costs would include improving the yield of fermentation, as well as increasing the volumetric rate of production by at least 2 fold (Bafana and Pandey, 2018). Recent efforts have been aimed at engineering organisms beyond *A. terreus* including *U. maydis, Y. lipolytica*, and *E. coli* (Krull et al., 2017b; Tehrani et al., 2019; Zhao et al., 2019).

### Mesaconic Acid

Lastly, another attractive potential route to MA is through mesaconic acid. Mescaconic acid is produced from the amino acid glutamate. Glutamic acid production *via* fermentation is a mature technology, primarily reliant on engineered *Corynebacterium glutamicum* (Kimura, 2003; Wendisch et al., 2016). Mesaconate production relies on several natural pathways for glutamate catabolism and/or carbon fixation (Figure 1N), wherein a mutase converts glutamate to methyl aspartate, which through the action of a methyl aspartase is converted to mesaconate (Wang and Zhang, 2015). Similarly to the HIBA-CoA mutase described above, the glutamate mutase is also a B12 dependent enzyme reliant on free radical chemistry and requires vitamin supplementation and continuous enzyme reactivation (Chih and Marsh, 2000; Wang and Zhang, 2015). Methyl aspartase shares a reaction mechanism with aspartases converting aspartate to fumarate (de Villiers et al., 2012). Heterologous expression of these enzymes from *C. tetanomorphum* in *E. coli* enabled mesaconic acid titers approaching 23 g/L (Wang and Zhang, 2015; Wang et al., 2018). The same basic chemical conversions producing MA from citramalate can convert mesaconic acid to MA although yields still require optimization and possible catalyst/process development (Johnson et al., 2012). While glutamate is basically a commodity chemical in its own right with prices estimated from $1.70–$1.95/kg ($2.00–2.25/kg of monosodium glutamate)^3^, assuming a yield of MA from glutamate of 0.58g/g, one could predict a potential MA cost of ~$2.90/kg which is not competitive with current pricing for MA. Cost reductions in glutamate production will be needed for this route to be competitive.

## Future Outlook

We can, admittedly subjectively, assess the relative maturity and remaining technical challenges for each of the proposed fermentation based routes to MA, as given in Table 1. In our opinion, the three most promising routes, when considering both the strain and bioprocess development, as well as final chemical conversions, are the routes through itaconic, citramalic, and isobutyric acids. All of these routes are well past proof of concept stages, but still will require optimization.

**Table 1:** Comparison of maturity and challenges for biobased routes to MA.

**Maturity**	**Route**	**Best demonstrated performance**	**Bioprocess challenges**	**Chemistry challenges**
1	Itaconic acid/decarboxylation	220g/L (*U. maydis*) (Tehrani et al., 2019) 0.45 g/L-h 51% bioprocess yield 48% conversion to MA (Johnson et al., 2009, 2012; Pirmoradi and Kastner, 2017)	Fermentation rates & yields	Yield, catalyst costs
2	Citramalic acid/decarboxylation & dehydration	80g/L (*E. coli*) (Webb et al., 2018) 1.85 g/L-h 58% bioprocess yield 81% conversion to MA (Johnson et al., 2009, 2012; Pirmoradi and Kastner, 2017)	Fermentation rates & yields	Yield, catalyst development
3	Isobutyric acid/dehydrogenation	90g/L (*E. coli*) (Xiong et al., 2015) 0.625 g/L-h 80% bioprocess yield 40–60% conversion to MA (Pirmoradi and Kastner, 2017)	Fermentation rates & yields	Catalyst development
4	2-HIBA/dehydration	6.4g/L (*C. necator H16)* (Hoefel et al., 2010) 0.09 g/L-h 6.3% bioprocess yield 71.5% conversion to MA (Pirmoradi and Kastner, 2017)	Enzymology	Yield, catalyst development
5	Mesaconic acid/decarboxylation	23g/L (*E. coli*) (Wang et al., 2018) 0.36 g/L-h 64% bioprocess yield 52% conversion to MA (Pirmoradi and Kastner, 2017)	Enzymology	Yield, catalyst development
6	Methacrylic acid production	0.0146g/L (*E. coli*) (Eastham et al., 2018) 0.0007 g/L-h 0.62% bioprocess yield	Rates, yields, engineering resistance	NA

While Table 1 has attempted to rank order the maturity of possible biobased routes to MA, all of the potential biological routes for MA from sugar, as described above, have a similar yield, 1 mole of MA from 1 mole of hexose, which translates in the case of glucose to a maximal theoretical yield of 0.477 gram of MA per gram of glucose. This yield is a common challenge to all of these routes. At theoretical yield, and glucose costs of $0.20/lb ($0.44/kg)^6^^,^^7^ this translates to a cost of $0.92/kg of MA for feedstock alone. This represents a major challenge to these biological routes, wherein they not only need to compete with the ACH manufacturing technology, but also newer ethylene based processes. In the case of the ACH process, converting acetone ($0.94/kg), HCN ($0.66/kg) and sulfuric acid ($0.13/kg) to MMA, we can estimate the cost of feedstocks for MA (which is not isolated as an intermediate in this process) at roughly $1/kg^3^. In this case, on a feedstock cost basis, glucose based routes have the potential to compete. However, the *greener* petroleum based routes converting ethylene ($0.76/kg), syngas (CO/H_2_, $0.07/kg), and methanol ($0.37/kg) or formaldehyde ($0.63/kg) to MA (Figures 1A,E), would have estimated costs for feedstocks of only $0.50/kg of MA^3^. This is half that of biobased routes, discussed in Figure 1.

**Figure 2:**
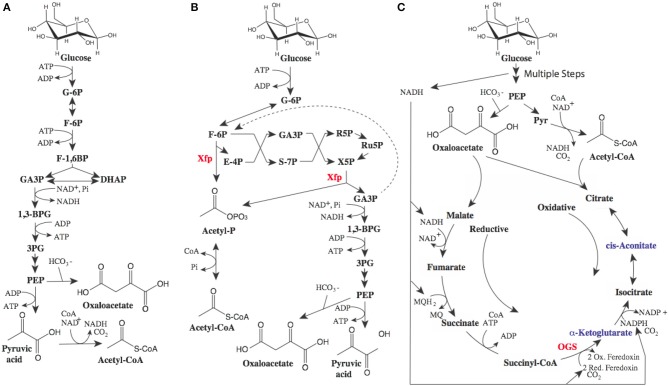
Potential metabolic pathways to optimize MA yields. **(A)** glycolytic metabolism, **(B)** Bifidobacterium shunt, and **(C)** reductive TCA cycle. In glycolysis **(A)** 0.5 moles of glucose are converted to 1 mole of pyruvate (or alternatively 1 mole of oxaloacetate) and one mole of NADH (1 pair or electrons). Pyruvate can be oxidized to acetyl-CoA generating another mole of NADH. **(B)** The Bifidobacterium shunt phosphoketolase enzyme (xfp) (Fandi et al., 2001) has activity as both an erythrose-4-phosphate (E4P) and xylulose-5-phosphate (X5P) phosphoketolase producing acetyl-phosphate (acetyl-P). Recycling 2 moles of glyceraldehyde-3-phosphate (GA3P) through triosephosphate isomerase and the reversible fructose bisphosphate aldolase can lead to improved yield (dashed line) **(C)**. Inclusion of 2-oxoglutarate synthase (OGS) in anaerobic production could lead metabolism where oxidative flux through the TCA cycle is balanced by reductive flux wherein electrons from glycolysis are consumed. Balanced TCA flux can lead to higher yields of alpha-ketoglutarate derived products such as mesaconic acid as well as cis-aconitate derived products such as itaconic acid. Additional abbreviations: glucose-6-phosphate (G-6P), fructose-6-phosphate (F-6P), fructose-1,6-bisphosphate (F-1,6BP), dihydroxyacetone phosphate (DHAP), 1,3 bisphosphoglycerate (1,3BPG), 3 phosphoglycerate (3PG), phosphoenolpyruvate (PEP).

While the proposed routes to MA have a maximal yield of 0.477 gram of MA per gram of glucose, there is room for improvement. The theoretical yield for MA from glucose is ~0.63g/g. A key limitation of these pathways is the wasting of electrons, as illustrated in Figure 2. While we often use the term “renewable carbon,” it is in actuality usually renewable reducing equivalents or electrons which are of the most value. The current biobased routes to MA described above all produce excess electrons. These electrons need to be oxidized for metabolism and production to proceed, either aerobically using oxygen, or anaerobically with another electron acceptor requiring the committed formation of unwanted fermentation byproducts. In addition, wasted pairs of electrons are accompanied by wasted carbon.

Fortunately, potential metabolic solutions to improve yields have been described, generally relying on the use of multiple metabolic routes in combination. For example, the combination of oxidative routes (described above) where excess electrons are generated and reductive routes where excess electrons can be utilized, can lead to improved yields. A good example of an oxidative route to the intermediate acetyl-CoA would be the bifidobacterium shunt, or non-oxidative glycolysis, as depicted in Figure 2B (Bogorad et al., 2013; Lin et al., 2018). This metabolic shunt has the potential to produce acetyl-CoA while conserving electrons and carbon, increasing maximal yields. In the best case (where some of the three carbon intermediates can be recaptured in the oxidative pathway, dashed line Figure 2B), maximal yields of MA reach theoretical yields of 0.63g/g. Non-oxidative glycolysis is useful for the routes to MA utilizing acetyl-CoA or pyruvate and acetyl-CoA (Figure 1). When evaluating the routes reliant on tricarboxylic acid (TCA) cycle intermediates or derivatives, consuming excess electrons produced *via* glycolysis in a reductive route can increase yields as demonstrated in Figure 2C. This would require expression of key enzymes from a natural reductive TCA cycle including 2-oxoglutarate synthase [OGS, which it should be noted is an oxygen sensitive enzyme (Hughes et al., 1998)]. A similar approach was used to increase 1,4-butanediol yield from TCA intermediates, albeit not requiring OGS (Yim et al., 2011). Again, this metabolism has the potential to increase theoretical yields of MA (from the mesaconic or itaconic acid intermediates) to the theoretical maximum of 0.63g/g.

With maximal yields of 0.63g/g, sugar costs of less than $0.32/kg ($0.145/lb) would still be needed for biobased routes to have feedstock costs comparable to newer ethylene based petrochemical processes. Sugar costs are a challenge in the bioeconomy in general, particularly for biobased commodities and especially for biofuels (Chen et al., 2013; NREL, 2013; Taylor et al., 2015; Rosales-Calderon and Arantes, 2019). These costs may well be achievable with second generation cellulosic based sugars as technologies for their production mature (Youngs and Somerville, 2012; Kühner, 2013; Liu et al., 2019). Previous estimates suggest cellulosic sugars can reach costs as low as $0.26/kg (Soare, 2013). Future changes in the legislative landscape, including potential carbon taxes or fines, may also help biobased routes compete (Rajni et al., 2006; Mustafa and Balat, 2009; Information Technology and Innovation Foundation, 2018; EPA, 2019). However, it is likely that technical developments to increase yields (as well as rates and titers), lower sugar costs, and legislative changes will be required for any potential biobased process to MA or MMA to take hold in the market.

## Author Contributions

JE extracted 10 year average chemical prices from the United States International Trade Commission (https://dataweb.usitc.gov/). JL, JE, and ML wrote, revised, and edited the manuscript.

### Conflict of Interest

ML has a financial interest in DMC Biotechnologies, Inc. The remaining authors declare that the research was conducted in the absence of any commercial or financial relationships that could be construed as a potential conflict of interest.

## Footnotes

^1^PMMA Market Size Worth $8.16 Billion By 2025|CAGR: 8.4%. https://www.grandviewresearch.com/press-release/global-polymethyl-methacrylate-pmma-industry.

^2^PMMA Production, Price and Market Demand. Plastics Insight https://www.plasticsinsight.com/resin-intelligence/resin-prices/pmma/.

^3^https://dataweb.usitc.gov/

^4^https://www.plasticsinsight.com/resin-intelligence/resin-prices/pmma/ and https://www.grandviewresearch.com/press-release/global-polymethyl-methacrylate-pmma-industry

^5^*Synthetic and Bio PMMA Market Size - Industry Share Report 2022*. Global Market Insights, Inc. https://www.gminsights.com/industry-analysis/synthetic-and-bio-based-pmma-polymethyl-methacrylate-market-size.

^6^*The Sweet and Sour History of Sugar Prices|Winton*. Winton https://www.winton.com/longer-view/the-sweet-and-sour-history-of-sugar-prices.

^7^Sugar Prices - 37 Year Historical Chart. https://www.macrotrends.net/2537/sugar-prices-historical-chart-data.
